# Evaluation of a candidate breast cancer associated SNP in *ERCC4* as a risk modifier in *BRCA1* and *BRCA2* mutation carriers. Results from the Consortium of Investigators of Modifiers of *BRCA1/BRCA2* (CIMBA)

**DOI:** 10.1038/sj.bjc.6605416

**Published:** 2009-11-17

**Authors:** A Osorio, R L Milne, G Pita, P Peterlongo, T Heikkinen, J Simard, G Chenevix-Trench, A B Spurdle, J Beesley, X Chen, S Healey, KConFab, S L Neuhausen, Y C Ding, F J Couch, X Wang, N Lindor, S Manoukian, M Barile, A Viel, L Tizzoni, C I Szabo, L Foretova, M Zikan, K Claes, M H Greene, P Mai, G Rennert, F Lejbkowicz, O Barnett-Griness, I L Andrulis, H Ozcelik, N Weerasooriya, OCGN, A-M Gerdes, M Thomassen, D G Cruger, M A Caligo, E Friedman, B Kaufman, Y Laitman, S Cohen, T Kontorovich, R Gershoni-Baruch, E Dagan, H Jernström, M S Askmalm, B Arver, B Malmer, SWE-BRCA, S M Domchek, K L Nathanson, J Brunet, T Ramón y Cajal, D Yannoukakos, U Hamann, HEBON, F B L Hogervorst, S Verhoef, EB Gómez García, J T Wijnen, A van den Ouweland, EMBRACE, D F Easton, S Peock, M Cook, C T Oliver, D Frost, C Luccarini, D G Evans, F Lalloo, R Eeles, G Pichert, J Cook, S Hodgson, P J Morrison, F Douglas, A K Godwin, GEMO,,, O M Sinilnikova, L Barjhoux, D Stoppa-Lyonnet, V Moncoutier, S Giraud, C Cassini, L Olivier-Faivre, F Révillion, J-P Peyrat, D Muller, J-P Fricker, H T Lynch, E M John, S Buys, M Daly, J L Hopper, M B Terry, A Miron, Y Yassin, D Goldgar, Breast Cancer Family Registry, C F Singer, D Gschwantler-Kaulich, G Pfeiler, A-C Spiess, Thomas v O Hansen, O T Johannsson, T Kirchhoff, K Offit, K Kosarin, M Piedmonte, G C Rodriguez, K Wakeley, J F Boggess, J Basil, P E Schwartz, S V Blank, A E Toland, M Montagna, C Casella, E N Imyanitov, A Allavena, R K Schmutzler, B Versmold, C Engel, A Meindl, N Ditsch, N Arnold, D Niederacher, H Deißler, B Fiebig, R Varon-Mateeva, D Schaefer, U G Froster, T Caldes, M de la Hoya, L McGuffog, A C Antoniou, H Nevanlinna, P Radice, J Benítez

**Affiliations:** 1 Human Genetics Group, Spanish National Cancer Research Centre, Madrid, Spain; 2 Genetic and Molecular Epidemiology Group, Spanish National Cancer Research Centre, Madrid, Spain; 3 Genotyping Unit, Human Cancer Genetics Programme, Spanish National Cancer Research Centre, Madrid, Spain; 4 Fondazione IRCCS Istituto Nazionale Tumori, Milan, Italy; 5 Fondazione Istituto FIRC di Oncologia Molecolare, Milan, Italy; 6 Department of Obstetrics and Gynaecology, Helsinki University Central Hospital (HUCH), HelsVinki, Finland; 7 Canada Research Chair in Oncogenetics, Cancer Genomics Laboratory, Centre Hospitalier Universitaire de Québec and Laval University, Québec, Canada; 8 Division of Genetics and Population Health, Queensland Institute of Medical Research, Brisbane, Australia; 9 Research Division, Peter MacCallum Cancer Center, Melbourne, Australia; 10 Department of Epidemiology, School of Medicine, University of California Irvine, Irvine, CA, USA; 11 Department of Laboratory Medicine and Pathology, Mayo Clinic, Rochester, MN, USA; 12 Department of Health Sciences Research, Mayo Clinic, Rochester, MN, USA; 13 Department of Medical Genetics, Mayo Clinic, Rochester, MN, USA; 14 Istituto Europeo di Oncologia, Milan, Italy; 15 Centro di Riferimento Oncologico, IRCCS, Aviano, PN, Italy; 16 Cogentech, Consortium for Genomic Technologies, Milan, Italy; 17 Department of Laboratory Medicine and Pathology, Mayo Clinic College of Medicine, Rochester, MN, USA; 18 Department of Cancer Epidemiology and Genetics, Masaryk Memorial Cancer Institute, Brno, Czech Republic; 19 Department of Biochemistry and Experimental Oncology, First Faculty of Medicine, Charles University, Prague, Czech Republic; 20 Center for Medical Genetics, Ghent University Hospital, Ghent, Belgium; 21 Clinical Genetics Branch, National Cancer Institute, Rockville, MD, USA; 22 CHS National Cancer Control Center at Carmel Medical Center and Technion Faculty of Medicine, Haifa, Israel; 23 Ontario Cancer Genetics Network (OCGN), Cancer Care Ontario, Toronto, ON, Canada; 24 Fred A. Litwin Center for Cancer Genetics, Samuel Lunenfeld Research Institute, Mount Sinai, Toronto, ON, Canada; 25 Clinical Genetics BFG, Odense University Hospital, Odense, Denmark; 26 Department of Clinical Genetics, Vejle Hospital, Vijle, Denmark; 27 Division of Pathology, Department of Oncology, University and University Hospital of Pisa, Pisa, Italy; 28 Sheba Medical Centre (SMC), Tel Hashomer, Israel; 29 The Sackler School of Medicine, Tel-Aviv University, Tel-Aviv, Israel; 30 Rambam Medical Centre, Haifa, Israel; 31 Department of Nursing, Faculty of Social Welfare and Health Sciences, University of Haifa, Haifa, Israel; 32 Rambam Health Care Campus, Institute of Human Genetics, Haifa, Israel; 33 Department of Oncology, Clinical Sciences, Lund University, Lund, Sweden; 34 Department of Oncology, University Hospital, Linköping, Sweden; 35 Department of Oncology, Karolinska University Hospital, Stockholm, Sweden; 36 Department of Radiation Sciences, Oncology, Umeå University, Umeå, Sweden; 37 See acknowledgements; 38 Abramson Cancer Center, University of Pennsylvania, Philadelphia, PA, USA; 39 Programa de Consejo Genético en Cáncer, Instituto Catalán de Oncología, Hospital Dr Josep Trueta-IdiBGi, Gerona, Spain; 40 Servicio de Oncología Médica del Hospital de la Santa Creu i Sant Pau, Barcelona, España; 41 IRRP, National Centre for Scientific Research ‘Demokritos’, Molecular Diagnostics Laboratory, Athens, Greece; 42 Molecular Genetics of Breast Cancer, Deutsches Krebsforschungszentrum (DKFZ), Heidelberg, Germany; 43 Department of Clinical Molecular Genetics, Family Cancer Clinic, Netherlands Cancer Institute, Amsterdam, NL, USA; 44 Department of Genetics and Cell Biology, University Medical Centre, Maastricht, NL; 45 Research Institute Growth and Development (GROW), Maastricht, NL, USA; 46 Department Clinical Molecular Genetics, Leiden University Medical Center, Leiden, NL, USA; 47 Department of Human Genetic, Leiden University Medical Center, Leiden, NL, USA; 48 Department of Molecular Genetics, Erasmus University Medical Center, Rotterdam, NL, USA; 49 Department of Public Health and Primary Care, Cancer Research UK Genetic Epidemiology Unit, University of Cambridge, Cambridge, UK; 50 Department of Oncology, University of Cambridge, Cambridge, UK; 51 Academic Unit of Medical Genetics and Regional Genetics Service, St Mary's Hospital, Manchester, UK; 52 Translational Cancer Genetics Team, The Institute of Cancer Research and Royal Marsden NHS Foundation Trust, London, UK; 53 Clinical Genetics, Guy's Hospital, London, UK; 54 Sheffield Clinical Genetics Service, Sheffield Children's Hospital, Sheffield, UK; 55 Department of Cancer Genetics, St Georges Hospital, University of London, London, UK; 56 Northern Ireland Regional Genetics Centre, Belfast City Hospital, Belfast, UK; 57 Institute of Human Genetics, Centre for Life, Newcastle Upon Tyne Hospitals NHS Trust, Newcastle upon Tyne, UK; 58 Fox Chase Cancer Center, Philadelphia, PA, USA; 59 Unité Mixte de Génétique Constitutionnelle des Cancers Fréquents, Hospices Civils de Lyon/Centre Léon Bérard, Lyon, France; 60 Laboratoire de Génétique Moléculaire, Signalisation et Cancer, UMR5201 CNRS, Université de Lyon, Lyon, France; 61 INSERM U509, Service de Génétique Oncologique, Institut Curie, Université Paris-Descartes, Paris, France; 62 Centre de Génétique, Dijon, France; 63 CLCC Georges François Leclerc, Dijon, France; 64 Human Molecular Oncology Laboratory, Centre Oscar Lambret, Lille, France; 65 Unité d’Oncogénétique, CLCC Paul Strauss, Strasbourg, France; 66 Department of Preventive Medicine, Creighton University, Omaha, NE, USA; 67 Northern California Cancer Center, Fremont, CA, USA; 68 Huntsman Cancer Institute, Salt Lake City, UT, USA; 69 Fox Chase Cancer Center, Philadelphia, PA, USA; 70 University of Melbourne, Melbourne, Australia; 71 Columbia University New York, NY, USA; 72 Dana Farber Cancer Institute, Boston, MA, USA; 73 Department of Dermatology, University of Utah, Salt Lake City, UT, USA; 74 Division of Special Gynecology, Department of OB/GYN, Medical University of Vienna, Vienna, Austria; 75 Department of Biochemistry, Pharmacology, and Genetics, Odense University Hospital, Odense, Denmark; 76 Department of Oncology, Landspitali-University Hospital, Reykjavik, Iceland (OThJ); 77 Department of Medicine, Clinical Genetics Service, Memorial Sloan-Kettering Cancer Center, New York, NY, USA; 78 GOG Statistical and Data Center, Roswell Park Cancer Institute, Buffalo, NY, USA; 79 NorthShore University Health System, Evanston Northwestern Healthcare, Evanston, IL, USA; 80 Tufts University, New England Medical Center, Boston, MA, USA; 81 University of North Carolina, Chapel Hill, NC, USA; 82 St Elizabeth Medical Center, Edgewood, KY, USA; 83 Yale University School of Medicine, New Haven, CT, USA; 84 New York University School of Medicine, New York, NY, USA; 85 Division of Human Cancer Genetics, Departments of Internal Medicine and Molecular Virology, Immunology and Medical Genetics, Comprehensive Cancer Center, The Ohio State University, Columbus, OHIO, USA; 86 Instituto Oncologico Veneto – IRCCS, Padua, Italy; 87 Department of Oncology and Surgical Sciences, Padua, Italy; 88 N.N. Petrov Institute of Oncology, St-Petersburg, Russia; 89 Department of Genetics, Biology and Biochemistry, University of Turin, Turin, Italy; 90 Division of Molecular Gynaeco-Oncology, Department of Obstetrics and Gynaecology, University of Cologne, Cologne, Germany; 91 Institute for Medical Informatics, Statistics and Epidemiology, University of Leipzig, Germany; 92 Department of Obstetrics and Gynaecology, Technical University Munich, Munich, Germany; 93 Department of Obstetrics and Gynaecology, Ludwig-Maximilian University Munich, Munich, Germany; 94 Department of Obstetrics and Gynaecology, University of Schleswig-Holstein, Campus Kiel, Germany; 95 Department of Obstetrics and Gynaecology, Molecular Genetics Laboratory, University of Duesseldorf, Dusseldorf, Germany; 96 Department of Obstetrics and Gynaecology, University of Ulm, Ulm, Germany; 97 University of Regensburg, Institute of Human Genetics, Regensburg, Germany; 98 Institute of Human Genetics, Charité, Humboldt University, Berlin, Germany; 99 Institute of Human Genetics, University of Frankfurt, Frankfurt, Germany; 100 Institute of Human Genetics, University of Leipzig, Leipzig, Germany; 101 Hospital Clínico San Carlos, Madrid, Spain

**Keywords:** *BRCA1*, *BRCA2*, *ERCC4*, breast cancer

## Abstract

**Background:**

In this study we aimed to evaluate the role of a SNP in intron 1 of the *ERCC4* gene (rs744154), previously reported to be associated with a reduced risk of breast cancer in the general population, as a breast cancer risk modifier in *BRCA1* and *BRCA2* mutation carriers.

**Methods:**

We have genotyped rs744154 in 9408 *BRCA1* and 5632 *BRCA2* mutation carriers from the Consortium of Investigators of Modifiers of *BRCA1/2* (CIMBA) and assessed its association with breast cancer risk using a retrospective weighted cohort approach.

**Results:**

We found no evidence of association with breast cancer risk for *BRCA1* (per-allele HR: 0.98, 95% CI: 0.93–1.04, *P*=0.5) or *BRCA2* (per-allele HR: 0.97, 95% CI: 0.89–1.06, *P*=0.5) mutation carriers.

**Conclusion:**

This SNP is not a significant modifier of breast cancer risk for mutation carriers, though weak associations cannot be ruled out.

Germ-line mutations in the *BRCA1* and *BRCA2* genes confer a high lifetime risk of developing breast and other cancers. Estimates of the cumulative risk of breast cancer to age 70 years vary from 40% to 85% (Easton *et al*, 1995; Ford *et al*, 1998; Antoniou *et al*, 2003; Chen *et al*, 2006; Milne *et al*, 2008). Other genetic and/or environmental factors (modifiers) are likely to explain these differences, at least in part. Because of the large sample size required to identify such effects, few reliable associations have been reported to date, all coming from the Consortium of Investigators of Modifiers of BRCA1/2 (CIMBA) initiative, which was set up to provide large samples of *BRCA1* and *BRCA2* mutation carriers to reliably assess even modest associations with single nucleotide polymorphisms (SNPs) (Chenevix-Trench *et al*, 2007). Recently, CIMBA assessed three SNPs in the *FGFR2*, *TNRC9* and *MAP3K1* genes that had been previously found by a genome-wide association study to be associated with increased breast cancer risk for women in the general population (Easton *et al*, 2007). Consistent associations were found for *BRCA1* and/or *BRCA2* mutation carriers (Antoniou *et al*, 2008), indicating that SNPs involved in the susceptibility to develop breast cancer in the general population are good candidates to be tested as potential modifiers in *BRCA1* and *BRCA2* mutation carriers.

The *ERCC4* gene is involved in the nucleotide excision repair (NER) pathway, which has led to the investigation of its role in the susceptibility to develop different types of cancer including breast cancer (Garcia-Closas *et al*, 2006; Mechanic *et al*, 2006; Moreno *et al*, 2006; Kiyohara and Yoshimasu, 2007; Hooker *et al*, 2008; Smith *et al*, 2008). We previously reported that the minor G allele in a SNP on intron 1 of *ERCC4* (rs744154) was associated with protection from breast cancer in the general population (OR under a recessive model 0.61; *P*=0.0002) (Milne *et al*, 2006). On the basis of this finding, we aimed to assess *ERCC4*-rs744154 as a breast cancer risk modifier in *BRCA1* and *BRCA2* mutation carriers. The study was performed in two stages, the first comprising 837 mutation carriers from three CIMBA centres, and the second comprising 15 040 mutation carriers from all the CIMBA studies, including those used in stage I.

## Materials and methods

### Subjects

Eligible subjects were female carriers of deleterious mutations in *BRCA1* and *BRCA2* aged 18 years or older. Further details regarding eligibility and the information collected from subjects are described elsewhere (Antoniou *et al*, 2008). Subjects who reported having ethnicity other than White European were excluded. This gave a total of 15 040 female mutation carriers (9408 with mutations in *BRCA1* and 5632 with mutations in *BRCA2*), 8088 of whom had been diagnosed with breast cancer (4956 and 3132 with mutations in *BRCA1* and *BRCA2*, respectively). All carriers participated in clinical or research studies at the host institution under ethically approved protocols.

A total of 34 collaborating CIMBA studies, carried out in 18 countries, contributed genotype data for *ERCC4*-rs744154 to this study. Details of each study along with the number of samples included from each are provided in Table 1. Seven studies (CBCS, GOG, ILUH, MSKCC, NNPIO, OSUCCG and IOVHBOCS) had not participated in previous CIMBA collaborations (Antoniou *et al*, 2008). Subjects from the CNIO, HEBCS and MBCSG studies were used in the first stage and comprised 837 mutation carriers (469 in *BRCA1* and 368 in *BRCA2*). All 9408 *BRCA1* and 5632 *BRCA2* mutation carriers CIMBA subjects were included in the second stage.

### Genotyping

The genotyping platform used by each study is detailed in Table 1. For 11 studies, matrix-assisted laser desorption/ionization time of flight mass spectrometry (MALDI-TOF MS) was applied to determine allele-specific primer extension products using Sequenom's MassARRAY system and iPLEX technology (Sequenom, San Diego, CA, USA). The design of oligonucleotides was carried out according to the guidelines of Sequenom and performed using MassARRAY Assay Design software (version 3.1). One study determined genotypes by direct sequencing. Genotyping was carried out for the remaining studies by nuclease assay (Taqman). Taqman genotyping reagents were designed by Applied Biosystems (Foster City, CA, USA) (http://www.appliedbiosystems.com/) as Assays-by-Design. Genotyping was performed using the ABI PRISM 7900HT, 7700 or 7500 Sequence Detection Systems according to the manufacturer's instructions. All studies complied with CIMBA genotyping quality control (QC) standards (Antoniou *et al*, 2008).

### Statistical analysis

To test for departure from Hardy–Weinberg equilibrium, a single subject was randomly selected from each family and Pearson's *χ*^2^ Test (1 d.f.) was applied to genotypes from this sample set. The association of *ERCC4*-rs744154 with breast cancer risk was assessed by estimating hazard ratios (HR) and their corresponding 95% confidence intervals (CI) using weighted multivariable Cox proportional hazards regression with robust estimates of variance (Antoniou *et al*, 2005). For each mutation carrier, we modelled the time to diagnosis of breast cancer from birth, censoring at the first of the following events: bilateral prophylactic mastectomy, breast cancer diagnosis, ovarian cancer diagnosis, death and last date known to be alive. Subjects were considered affected if they were censored at breast cancer diagnosis and unaffected otherwise. The weighted cohort approach involves assigning weights separately to affected and unaffected individuals such that the weighted observed incidences in the sample agree with established estimates for mutation carriers (Antoniou *et al*, 2003). This approach has been shown to adjust for the bias in the HR estimates that is a consequence of the ascertainment criteria used (Antoniou *et al*, 2005), which leads to an over-sampling of affected women. Weights were assigned separately for carriers of mutations in *BRCA1* and *BRCA2* and by age interval (<25, 25–29, 30–34, 35–39, 40–44, 45–49, 50–54, 55–59, 60–64, 65–69, ⩾70).

We considered log-additive and codominant genetic models and tested these by applying in the first case a Wald test based on the log-HR estimate per allele and its standard error, and in the second the *χ*^2^ equivalent of a Wald test (on 2 degrees of freedom [d.f.]) based on the log-HR estimates for heterozygotes (CG) and minor-allele-homozygotes (GG) *vs* common homozygotes (CC) and the corresponding variance–covariance matrix. Additional independent variables included in all analyses were year of birth (<1930, 1930–1939, 1940–1949, 1950–1959, 1960–1969, ⩾1970), study centre and country. Heterogeneity in HRs by study centre was assessed by the *χ*^2^ test described above, but applied to interaction terms for the per-allele effect by centre (on 33 d.f.). A number of sensitivity analyses were applied, including censoring at bilateral prophylactic oophorectomy (BPO), adjusting for BPO (as a time-varying covariate), excluding data used in the initial analysis (from CNIO, HEBCS and MBCSG) and excluding prevalent cases, defined as those diagnosed >3 years before interview or DNA extraction. In addition, an alternative analysis based on a previously described retrospective likelihood approach (Chenevix-Trench *et al*, 2007) was also applied using the pedigree analysis software MENDEL (Lange *et al*, 1988).

All statistical analyses were carried out using Stata: Release 10 (StataCorp. 2007. Stata Statistical Software: Release 10.0. College Station, TX: Stata Corporation LP) unless otherwise stated. Robust estimates of variance were calculated using the *cluster* subcommand, applied to an identifier variable unique to each family.

## Results and discussion

It is thought that any SNP involved in the susceptibility to develop breast cancer in the general population could also be a phenotypic modifier in carriers of mutations in the high-risk susceptibility genes *BRCA1* and *BRCA2*. This has been confirmed in a recent report from CIMBA in which minor alleles in three SNPs in the *FGFR2*, *TNRC9* and *MAP3K1* genes, previously found to be associated with increased breast cancer risk in the general population (Easton *et al*, 2007), were found to increase breast cancer risk in *BRCA1* and/or *BRCA2* mutation carriers as well (Antoniou *et al*, 2008). We therefore aimed to investigate the role of the rs744154 SNP in *ERCC4* as a potential *BRCA1/BRCA2* risk modifier, based on our earlier finding that the minor G allele was associated with breast cancer protection in the general population (OR under a recessive model 0.61; *P*=0.0002) (Milne *et al*, 2006).

This study was performed in two stages, the first analysing the SNP in 837 carriers of mutations (469 in *BRCA1* and 368 in *BRCA2*) from three CIMBA studies (CNIO, HEBCS and MBCSG). Results of the first stage are summarized in Table 2. We observed a marginally significant association of the G allele in *ERCC4*-rs744154 with breast cancer risk for both *BRCA1* (HR: 0.78, 95% CI: 0.60–1.00, *P*=0.05) and *BRCA2* (HR: 0.68, 95% CI: 0.45–1.02, *P*=0.06) mutation carriers. As this result was consistent with our previously reported protective association in the general population (Milne *et al*, 2006), we genotyped this SNP in subjects from the remaining CIMBA studies and repeated the analysis in the combined series of 9408 *BRCA1* and 5632 *BRCA2* mutation carriers (see Table 2). However, when the whole CIMBA series was analysed (stage II), there was no longer evidence of an association with breast cancer risk for either *BRCA1* (HR: 0.98, 95% CI: 0.93–1.04, *P*=0.5) or *BRCA2* (HR: 0.97, 95% CI: 0.89–1.06, *P*=0.5) mutation carriers. Several sensitivity analyses were performed (see Materials and Methods) but results did not change substantially and so those from the main analysis are presented in this report.

Subsequent to the initiation of this study, a study from the Breast Cancer Association Consortium (BCAC), performed in >30 000 breast cancer cases and 30 000 controls has now found no evidence for an association of rs744154 with breast cancer risk in the general population (Gaudet *et al*, 2009). This is consistent with the lack of association found for *BRCA1* and *BRCA2* mutation carriers. These findings highlight the necessity of very large collaborative efforts to obtain reliable conclusions in genetic association studies. On the basis of the combined results obtained from the BCAC analysis of sporadic breast cancer and the CIMBA analysis of BRCA-related breast cancer – each the largest study of its kind – there no longer seems to be convincing evidence that *ERCC4*-rs744154 is associated with breast cancer risk.

## Figures and Tables

**Table 1:** Number of *BRCA1* and *BRCA2* mutation carriers by study

**Study**	**Country of residence**	***BRCA1***	***BRCA2***	**Genotyping platform**
Breast Cancer Family Registry (BCFR)	USA and Australia	492	356	Taqman
Copenhagen Breast Cancer Study (CBCS)	Denmark	92	51	Taqman
CNIO	Spain and Greece^a^	149	198	Taqman
Deutsches Krebsforschungszentrum (DKFZ)	Germany	68	27	Taqman
Hereditary Breast and Ovarian study Netherlands (DNA-HEBON)	The Netherlands	768	293	iPlex^b^
Epidemiological study of BRCA1 and BRCA2 mutation carriers (EMBRACE)	UK and Eire	801	616	iPlex^b^
Fox Chase Cancer Center (FCCC)	USA	82	53	iPlex^b^
German Consortium of Hereditary Breast and Ovarian Cancer (GC-HBOC)	Germany	799	376	Taqman
Genetic Modifiers of cancer risk in BRCA1/2 mutation carriers (GEMO)	France	1123	565	Taqman
Gynecologic Oncology group (GOG)	USA	391	275	Taqman
Georgetown (GTN)	USA	29	17	iPlex^b^
Hospital Clínico San Carlos (HCSC)	Spain	109	94	Taqman
Helsinki Breast Cancer Study (HEBCS)	Finland	102	104	iPlex^b^
Iceland Landspitali – University Hospital (ILUH)	Iceland		86	Sequencing
Interdisciplinary Health Research International Team Breast Cancer Susceptibility (INHERIT BRCAs)	Quebec-Canada	73	82	Taqman
Kathleen Cuningham Consortium for Research into Familial Breast Cancer (kConFab)	Australia	488	388	iPlex^b^
Mayo Clinic (MAYO)	USA	214	118	iPlex^b^
Milan Breast Cancer Study Group (MBCSG)	Italy	344	218	Taqman
Modifier Study of Quantitative Effects on Disease (ModSQuaD)	Czech Republic	271	128	Taqman
Memorial Sloan-Kettering Cancer Center (MSKCC)	USA	255	157	Taqman
Medical University of Vienna (MUV)	Austria	281	120	iPlex^b^
National Cancer Institute (NCI)	USA	156	73	Taqman
National Israeli Cancer Control Centre (NICCC)	Israel	309	196	Taqman
N.N. Petrov Institute of Oncology (NNPIO)	Russia	66	0	Taqman
Ontario Cancer Genetics Network (OCGN)	Canada	219	170	Taqman
The Ohio State University Clinical Cancer Genetics Program (OSU CCG)	USA	60	31	Taqman
Odense University Hospital (OUH)	Denmark	217	131	Taqman
Istituto Oncologico Veneto (IOVHBOCS)	Italy	93	88	Taqman
Pisa Breast Cancer Study (PBCS)	Italy	72	40	iPlex^b^
Sheba Medical Centre (SMC)-Tel Hashomer	Israel	400	190	Taqman
Swedish Breast Cancer Study (SWE-BRCA)	Sweden	411	120	iPlex^b^
University of Turín Breast Cancer Study (UTBCS)	Italy	61	43	Taqman
University of California Irvine (UCI)	USA	166	120	Taqman
University of Pennsylvania (UPENN)	USA	247	108	iPlex^b^
Total		9408	5632	

a The CNIO series consisted of mutation carriers from the Spanish Consortium for the Study of Genetic Modifiers of *BRCA1* and *BRCA2* and the NCSR Demokritos, Athens, Greece.

b Mutation carriers that failed genotyping are not included in the totals.

**Table 2:** Genotype frequencies of *ERCC4*-rs744154 by mutation and disease status and hazard ratio estimates from stages I and II

	**Genotype**	**Unaffected (%)**	**Affected (%)**	**HR^a^**	**95% CI**	***P*-value**
*Stage I* a						
*BRCA1* (*n*=469)	CC	104 (50)	148 (57)	1.00		
	CG	85 (41)	96 (37)	0.86	0.62–1.20	
	GG	19 (9)	17 (7)	0.49	0.24–1.01	0.1^b^
	Per allele			0.78	0.60–1.00	0.05
						
*BRCA2* (*n*=368)	CC	73 (45)	109 (53)	1.00		
	CG	81 (49)	82 (40)	0.66	0.40–1.09	
	GG	10 (6)	13 (6)	0.50	0.17–1.47	0.2^b^
	Per allele			0.68	0.45–1.02	0.06
						
*Stage II*						
*BRCA1* (*n*=9408)	CC	2251 (51)	2603 (53)	1.00		
	CG	1836 (41)	1922 (39)	0.99	0.92–1.07	
	GG	365 (8)	431 (9)	0.96	0.83–1.10	0.8^b^
	Per allele			0.98	0.93–1.04	0.5
						
*BRCA2* (*n*=5632)	CC	1288 (52)	1601 (51)	1.00		
	CG	1012 (40)	1300 (42)	1.05	0.93–1.18	
	GG	200 (8)	231 (7)	0.82	0.65–1.02	0.09^b^
	Per allele			0.97	0.89–1.06	0.5

a 837 mutation carriers from CNIO, MBCSG and HBCS were included in stage I. Additional mutation carriers from these centres were later genotyped and included in stage II, with carriers from other CIMBA centres. Total number of mutation carriers from these three centres included in the study is provided in Table 1.

b 2-d.f. test.
